# Beverages Based on Second Quality Citrus Fruits and Maqui Berry, a Source of Bioactive (Poly)phenols: Sorting Out Urine Metabolites upon a Longitudinal Study

**DOI:** 10.3390/nu13020312

**Published:** 2021-01-22

**Authors:** Vicente Agulló, Cristina García-Viguera, Raúl Domínguez-Perles

**Affiliations:** Phytochemistry and Healthy Foods Lab, Group of Quality, Safety, and Bioactivity of Plant Foods, Department of Food Science and Technology, (CEBAS-CSIC), University Campus of Espinardo, Edif. 25, 30100 Murcia, Spain; vagullo@cebas.csic.es (V.A.); rdperles@cebas.csic.es (R.D.-P.)

**Keywords:** dietary intervention, beverage, anthocyanins, flavanones, bioavailability, UHPLC-ESI-QqQ-MS/MS

## Abstract

The intake of sugar-sweetened beverages has been associated with an augmented prevalence of metabolic diseases, namely, obesity, type II diabetes, and metabolic syndrome. On the other hand, nowadays, it is broadly accepted that foods and beverages rich in (poly)phenols could contribute to reducing the incidence of these pathologies. In this sense, the objective of the work was to revalue second quality citrus fruits for the development of new beverages, rich in anthocyanins and flavanones (maqui berry and second qualities citrus-based), and evaluate the influence of alternative sweeteners (sucralose, sucrose, or stevia), regarding the bioaccessibility and bioavailability of these bioactive compounds in the frame of a chronic (longitudinal) intervention. To fulfill this objective, a longitudinal study of the urinary excretion of anthocyanins and flavanones, after 2-months of ingestion of the developed maqui-citrus beverage, by 138 volunteers (*n* = 46 per beverage) and the analysis of the resulting phenolic metabolites by ultra-high performance liquid chromatography coupled to mass spectrometry (UHPLC-ESI-QqQ-MS/MS) was carried out. As major results, the bioavailable metabolites of caffeic acid (CA), catechol (CAT), 3,4-di-hydroxyphenylacetic acid (DHPAA), eriodictyol (E), homoeriodictyol (HE), hippuric acid (HA), naringenin (N), trans-ferulic acid (TFA), 2,4,6-tri-hydroxybenzaldehyde (THBA), trans-isoferulic acid (TIFA), and vanillic acid (VA) were detected. Accordingly, significantly different bioavailability was dependent on the sweetener used, allowing proposing stevia and, to a lower extent, sucralose, as valuable alternatives to sucrose.

## 1. Introduction

The rising incidence of metabolic diseases, namely, obesity, metabolic syndrome, or type II diabetes mellitus, is one of the main social challenges ahead now and in the coming years [1]. These diseases are closely related to dietary habits, in general, and particularly associated with the consumption of sugar-sweetened beverages [2]. Moreover, the intake of this type of beverage has been associated with weight gain, hypertension, and cardiovascular diseases that could threaten the sustainability of the health systems [3,4,5].

To minimize the consumption of sugar-sweetened beverages, global trends, based on the use of alternative sweeteners towards healthy beverages, sources of dietary bioactive compounds, are focusing the attention of the research activity [6], because of the effect of replacing sugar with artificial sweeteners on the risk of developing chronic pathologies, such as cardiovascular diseases or type II diabetes mellitus, among others [7].

In parallel to sugar replacement strategies, fruit beverages/juices, which are rich in bioactive compounds with demonstrated health benefits, could therefore contribute to preventing the incidence and severity of those pathologies referred above, associated with sugar consumption [8]. Thereby, this fact has boosted the assessment of newly developed bioactive beverages based on fruits featured by a valuable content of (poly)phenols [9,10], which consumption entails a bioavailability of (poly)phenols ranging between 5 and 10%. However, it has to be noticed that these bioavailability ratios correspond to studies focused on non-metabolized compounds, while the values rise to 60–70% when considering also the formation of phase II metabolites. Anyway, the efficiency of intestinal absorption of bioactive (poly)phenols is mostly conditioned by the physicochemical properties of the diverse food/beverage matrices that influences the bioaccessibility of the compounds of interest, as well as the interindividual variability [11]. Despite this controversy, upon a study carried out by Ribas-Agusti et al. [12], positive interactions between sucrose and phenolic compounds have been suggested, which would inform on the relevance of sugar for establishing the actual (poly)phenols bioavailability. This fact has been partially explored by Agulló et al. [13,14], who reported stevia and sucralose as valuable alternatives to sucrose, in terms of bioavailability of phenolic compounds after an acute administration of a (poly)phenol-rich beverage. In this regard, the long-term intervention study described in the present work provides helpful information to augment the knowledge about the effect of stevia and sucralose on human health after long-term ingestion.

Maqui berry (*Aristotelia chilensis* (Mol.) Stuntz) is a purple blackberry from Chile and Argentina rich in anthocyanins (delphinidin and cyanidin derivatives) that has been broadly selected to develop healthy beverages, because of the biological attributions of its (poly)phenolic fraction (high antioxidant capacity, cardio-protection, and inhibition of adipogenesis and diabetes symptoms) [9]. On the other hand, citrus fruits are featured by an outstanding flavanone content, with naringenin, eriocitrin, and hesperidin being the most representative flavanones in these fruits. This composition has prompted the selection of, mainly lemon plus other, citrus fruits to design and develop new bioactive beverages [8,13,14,15]. Indeed, the phenolic composition described for citrus fruits has been associated with health benefits against cardiovascular disease, cancer, type II diabetes mellitus, and obesity [15,16]. The citrus, mainly lemon fruit, production entails a high proportion of by-products represented by second quality fruits featured by a lower appearance quality that cannot be marketed for fresh consumption, being required to envisage additional applications that would contribute to the sustainability of its production in the Mediterranean Basin [15,17]. In this regard, the use of the second quality citrus fruits in the development of new potentially bioactive beverages could be a valuable approach to take advantage of a second quality product that does not allow their commercialization for fresh consumption. However, the identification of valorization alternative as a source of dietary bioactive (poly)phenols provides an especially appropriate use, allowing obtaining new added-value beverages, as its processed juice is not very appreciated, due to its high acidity.

Taking into account that background and following previous researches [13,14], the present work deals with the relationship between sweeteners and bioavailability of phenolic compounds, especially flavanones and anthocyanins, on healthy overweighed humans, after a chronic intake of maqui-citrus drinks with different sweeteners: sucrose (natural high caloric), stevia (natural non-caloric), and sucralose (artificial non-caloric). This approach would allow us a new advance, monitoring the possible accumulative effect of these (poly)phenols, as well as valorizing second qualities citrus, not allowed for fresh for fresh consumption.

## 2. Materials and Methods

### 2.1. Chemicals and Reagents

Cyanidin (Cy) 3-O-glucoside, delphinidin (Dp) 3-O-glucoside, eriodictyol (E), homoeriodictyol (HE), naringenin, and hesperetin were purchased from TransMIT (Geiben, Germany). Hesperidin, eriocitrin, and naringenin were obtained from Merck (Darmstadt, Germany). Caffeic (CA; also known as 3,4-di-hydroxycinnamic acid), gallic (GA; also known as 3,4,5-tri-hydroxybenzoic acid), 3,4-di-hydroxyphenylacetic (DHPAA), hippuric (HA), trans-ferulic (TFA; also known as 4-hydroxy-3-methoxycinnamic acid), trans-isoferulic (TIFA; also known as 3-hydroxy-4-methoxycinnamic acid), and vanillic (VA; also known as 4-hydroxy-3-methoxybenzoic acid) acids, 2,4,6-tri-hydroxybenzaldehyde (THBA), and catechol (CAT; also known as benzene-1,2-diol) were obtained from Sigma-Aldrich (Steinheim, Germany). Formic acid and acetonitrile of analytical grade were obtained from Fisher-Scientific (Loughborough, UK). All solutions were prepared with ultrapure deionized water from a Milli-Q Advantage A10 ultrapure water purification system (Millipore, Burlington, MA, USA).

### 2.2. Beverages Preparation and Characterization of the Phenolic Content

Maqui New Life S.A. (Santiago, Chile), and Cítricos de Murcia S.L. (Ceutí, Spain) and AMC Grupo Alimentación Fresco y Zumos S.A. (Espinardo, Spain) provided fresh dry organic maqui powder and the citrus juices, respectively. Sucrose, Stevia, and sucralose were provided by AB Azucarera Iberia S.L. (Madrid, Spain), AgriStevia S.L. (Molina de Segura, Spain), and Zukan (Murcia, Spain), respectively.

Maqui-citrus beverages’ (maqui plus mainly second quality lemon together with other citrus juices) performance was according to the previous description in the bibliography [18]. Briefly, maqui powder was mixed with juices elaborated from second quality citrus fruits (lemon juice mixed with other citrus in a certain proportion, in order to reduce acidity and obtain a more acceptable beverage) to obtain the base drink. Afterward, sweeteners were added in different proportions, stevia 4 mg per 100 mL and sucrose 7.5 g per 100 mL, to obtain similar acceptable sweetness in all beverages. Each different sweetener beverage (400 L) was pasteurized according to common industrial standards (85 °C for 15 s) for citrus drinks and pH at the Universidad Miguel Hernández (Orihuela, Alicante, Spain, under the supervision of Dr Martí) and bottled (330 mL volume bottles). This was repeated every 15 days, as the drinks were provided in lots of 15 bottles, for their intake in the following 15 days, with instructions for preserving them in the fridge. In this way, the freshness of the ready-to-drink beverages was preserved. The type of sweetener was unknown for volunteers, researchers (who provided the beverages, and collected/processed the samples), as well as for researchers that developed the statistical analyses. The phenolic composition of the beverages developed was studied following the methodology previously described [18,19]. The proximate composition of the juices was also determined (Table S1). Moreover, quality and safety tests were performed, as well as shelf-life tests, which confirmed that the beverages were innocuous from the toxicological and microbiological point of view, while the nutritional and phytochemical composition remained intact [18].

### 2.3. Experimental Design

A double-blind, randomized, longitudinal, crossover clinical study was performed in overweight individuals (*n* = 138), by the Catholic University of Murcia (UCAM, Murcia, Spain), under the supervision of Dr. Villaño. The study was conducted under the principles of the Declaration of Helsinki, and the protocol was approved by the Official Ethical Committee of Clinical Studies (CEIC) of University Hospital “Morales Meseguer” (Murcia, Spain) and registered at ClinicalTrials.gov (NCT04016337). Overweight volunteers (BMI: ~28) were provided with written information about the study and all of them signed the informed consent. The criteria for volunteers’ selection to participate in the study were as followed in previous studies [13,14]. The intervention study consisted of the daily ingestion of the maqui-citrus beverage (330 mL), over 60 days. Urine samples were collected at the initial (day 0) and final days of intervention (day 60) and were stored at −80 °C until analyses that were performed once the intervention period was finished and in the same batch to minimize analytical variations.

### 2.4. Urine Samples Collection, Processing, and Analysis by UHPLC-ESI-QqQ-MS/MS

Urine samples were processed following the method previously described [13,14]. The identification and quantification of phenolic metabolites were performed by applying the method previously reported [13,14].

### 2.5. Statistical Analysis

Quantitative data are presented as mean ± SD of 46 volunteers. Specific differences between basal and final concentrations were examined by a paired *t-*test based on their normal distribution. The data were processed using the SPSS 25.0 software package (SPSS Inc., Chicago, IL, USA) and the level of significance was set at *p* < 0.05.

## 3. Results

### 3.1. (Poly)phenolic Content of Beverages

The assessment of the beverages performed using three different sweeteners (stevia, sucrose, and sucralose) on their (poly)phenolic quantitative profile allowed identifying their content of individual flavanones and anthocyanins. In this regard, it was observed the presence, in the drinks, of four flavanones, which were found in the following decreasing concentration order, hesperidin (hesperetin 7-O-rutinoside) > eriocitrin (eriodictyol 7-O-rutinoside) > narirutin (naringenin 7-O-rutinoside) > O-tri-glycosyl-naringenin (Table 1).

On the other hand, regarding anthocyanins, it was found Dp 3,5-O-di-glc > Dp 3-O-sam-5-O-glc ≈ Dp 3-O-glc > co-eluting Cy 3-O-sam-5-O-glc and Cy 3,5-O-di-glc > Dp 3-O-sam > Cy 3-O-glc > Cy 3-O-sam (Table 2). When comparing the quantitative profile of the separate drinks, no statistically significant differences (*p >* 0.05) were observed between the concentration of the flavanones and anthocyanins identified in the separate maqui-citrus drinks, as a consequence of the sweetener applied, concerning both individual and total flavanones and anthocyanins (Table 1 and Table 2).

This (poly)phenolic content is, to some extent, in agreement with the drinks developed by Gironés et al., being the differences found probably due to the diverse formulations of the beverages used in both studies. In this regard, the beverages described by Girones et al. were characterized by the antioxidant capacity, as well as lipase and α-glucuronidase inhibitory activity, demonstrating an important role in the prevention of obesity and type II diabetes mellitus, among other metabolic disorders [9,19].

### 3.2. Characterization of the Urine Profile of Flavanones and Anthocyanins Ingested by Maqui-Citrus Beverages

The assessment of the urine profile of flavanones and anthocyanins present in the maqui-citrus beverages, which were absorbed at the intestinal level, metabolized, and excreted by the urine, was done to identify differences tentatively attributable to the sweetener employed (stevia, sucralose, and sucrose). Thereby, it was applied a targeted metabolomic approach for the identification of the 36 (poly)phenolic urine metabolites described in previous acute intervention studies (Complementary data Table S2) [13,14]. Specifically, from the range of the target (poly)phenolic derivatives monitored, the compounds identified, in the present chronic intervention study, were caffeic acid (CA), CA glucuronide, CA sulfate, CA glucuronide-sulfate, catechol (CAT) sulfate, 3,4-di-hydroxyphenylacetic acid (DHPAA), DHPAA glucuronide, DHPAA di-glucuronide, DHPAA glucuronide-sulfate, DHPAA di-sulfate, eriodictyol (E), E glucuronide, E sulfate, E di-sulfate, homoeriodictyol (HE), HE glucuronide, HE di-glucuronide, HE sulfate, hippuric acid (HA), HA sulfate, naringenin (N), N glucuronide, N di-glucuronide, N sulfate, N glucuronide-sulfate, trans-ferulic acid (TFA) glucuronide, TFA di-glucuronide, TFA sulfate, TFA di-sulfate, 2,4,6-tri-hydroxybenzaldehyde (THBA) glucuronide, trans-isoferulic acid (TIFA) sulfate, vanillic acid (VA), VA di-glucuronide, VA glucuronide-sulfate, and VA di-sulfate.

Interestingly, regarding the anthocyanins and hesperidin, their aglycones were not detected. Regarding anthocyanins, this fact could be attributed to the degradation under the physicochemical conditions featuring gastrointestinal digestion and/or their metabolism after absorption as a result of phase II metabolic reactions in the intestine epithelium and hepatocytes, forming glucuronide-, sulfate-, or methyl-derivatives in the proximal gastrointestinal tract [20]. Similarly, the lack of successful identification of hesperetin derivatives could be due to the participation of similar metabolic routes in the metabolism of flavanones. Besides, some phenolic metabolites referred to above were found in the urine of a reduced number of volunteers, specifically catechol sulfate, eriodictyol (E) di-sulfate, homoeriodictyol (HE) and its metabolites (HE di-glucuronide and HE sulfate), naringenin (N) glucuronide-sulfate, trans-ferulic acid (TFA) di-glucuronide, and 2,4,6-tri-hydroxybenzaldehyde (THBA) glucuronide. Indeed, these compounds were found in quantifiable concentrations in the urine of a limited number of volunteers that turns them into no representative. This fact may be due to interindividual variations regarding metabolic traits, also responsible, in the frame of the assessments described in the present work, for the dispersion featuring the concentrations of the metabolites identified in vivo [21]. On the other hand, caffeic acid (CA) and its metabolites (CA glucuronide, CA sulfate, and CA glucuronide-sulfate), 3,4-di-hydroxyphenylacetic acid (DHPAA) and its metabolites (DHPAA glucuronide, DHPAA di-glucuronide, DHPAA glucuronide-sulfate, and DHPAA di-sulfate), eriodictyol (E) and its metabolites (E glucuronide and E sulfate), homoeriodictyol (HE) glucuronide, hippuric acid (HA), and HA sulfate, naringenin (N) and its metabolites (N glucuronide, N di-glucuronide, and N sulfate), trans-ferulic acid (TFA) metabolites (TFA glucuronide, TFA sulfate, and TFA di-sulfate), trans-isoferulic acid (TIFA) sulfate, and vanillic acid (VA) and its metabolites (VA di-glucuronide, VA glucuronide-sulfate, and VA di-sulfate) were identified and quantified in the urine of all volunteers. Nevertheless, hippuric acid (HA) was not taken into consideration because of its high basal levels found as a result of its endogenous production, as well as the widespread occurrence of this compound in a broad diversity of dietary sources, which did not allow the quantification of the amount obtained from the metabolism of the (poly)phenols of the maqui-citrus beverages [22].

### 3.3. Urine Concentration of Flavanone and Anthocyanin Metabolites after Chronic Ingestion of Maqui-Citrus Beverages

**Figure 1:**
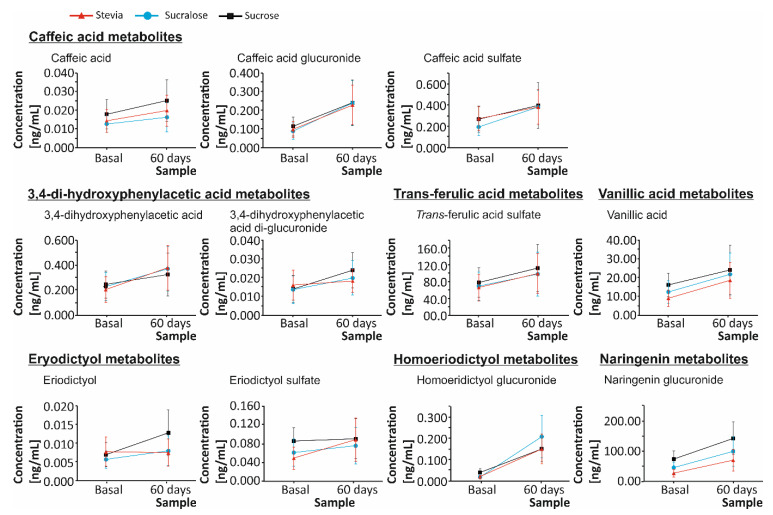
Content of single anthocyanins and flavanones metabolites (caffeic acid, caffeic acid glucuronide, caffeic acid sulfate, 3,4-di-hydroxyphenylacetic acid, 3,4-di-hydroxyphenylacetic acid di-glucuronide, eriodictyol, eriodictyol sulfate, homoeriodictyol glucuronide, naringenin glucuronide, trans-ferulic acid sulfate, and vanillic acid) in basal urine and 60 days urine of healthy volunteers after daily ingestion of 330 mL of maqui-citrus drinks developed using as sweeteners stevia (red ∆), sucralose (blue O), and sucrose (black □).

The quantification of the metabolites excreted in the urine was developed on basal urine (before the beginning of the dietary intervention) and the urine excreted after 60 days of daily ingestion of 330 mL of maqui-citrus beverage (Figure 1). The data retrieved evidenced that, although 27 metabolites were identified and quantified in the urine of all volunteers, exhibiting a rising trend relative to the concentration recorded in the basal urine. The differences found were statistically significant only for the 15 metabolites showed in Figure 1. Thus, although the dietary intervention set-up allowed increasing the bioavailability of the bioactive compounds present in the maqui-citrus drik, the absence of significant differences for some of them would be attributable to interindividual variations of the metabolic traits that give rise to the dispersion of data on the quantitative profile of urine metabolites. Indeed, this fact is a major constraint associated with the dietary intervention studies that would difficult the identification of the actual differences between basal and final urine concentration for the metabolites of interest.

#### 3.3.1. Caffeic Acid Derivatives

Regarding the metabolites consistently excreted by most volunteers, caffeic acid (CA) is a natural phenolic compound and a common degradation product of both flavanones and anthocyanins [22]. The bioavailability of CA involves a range of derivatives, namely, unesterified caffeic acid (CA), CA glucuronide, and CA sulfate, that, in the present work, showed a significant increase (*p* < 0.05) after 60 days consumption of the citrus-maqui-berry beverages, closely dependent on the sweetener applied (Figure 1). When analyzing the different concentrations obtained depending on the beverage ingested, the highest amount of the separate CA derivatives was achieved as a result of the intake of drinks developed using sucrose as a sweetener (0.03, 0.24, and 0.39 µg/mg of creatinine for unesterified CA, CA glucuronide, and CA sulfate, respectively). However, regarding unesterified caffeic acid (CA), beverages done using sucrose and stevia showed the highest percentage increase relative to the basal levels (41% and 39%, respectively). Concerning the glucuronide and sulfate derivatives of caffeic acid (CA), the beverage including sucralose as a sweetener provided a 34% and 55% higher increase, respectively, than stevia- and sucrose-based drinks, on average (Figure 1).

Concerning these metabolites, when applying a paired *t*-test to identify the statistically significant augments attained depending on the sweetener used in the development of the beverages, it was observed that the urine excretion of unesterified CA augmented significantly after the ingestion of stevia- and sucrose-based beverage (*p* < 0.01), as well as, to a lower extent, sucralose-based beverages (*p* < 0.05). Besides, for CA glucuronide, a significant augment (*p* < 0.001) was observed independently of the sweetener used. Regarding CA sulfate, sucralose-based drinks provided the most significant increase of the urine concentration (*p* < 0.001), followed by stevia- and sucrose-based beverages, which remained at a similar level (*p* < 0.05) (Figure 1). Furthermore, CA glucuronide-sulfate was not detected in the basal urine, but after the ingestion of the newly developed drinks, the highest values were reached due to the intake of the sucrose-sweetened beverage (0.003 µg/mg of creatinine). With respect to the biological attributions of bioavailable CA derivatives, to the present date, it has been reported several healthy properties, such as a protective activity against type II diabetes mellitus and angiopathy, along with valuable contributions to the regulation of gut microbiota [23,24]. In this regard, the augmented bioavailability of such metabolites, as a result of this dietary intervention, would connect the exploration of new sweeteners with a positive effect on health derived from the augmented bioavailability of bioactive (poly)phenols, beyond the only reduction of the sugar intake and its harmful metabolic effects.

#### 3.3.2. 3,4-Di-Hydroxyphenylacetic Acid Derivatives

Regarding 3,4-di-hydroxyphenylacetic acid (DHPAA), this is a microbiota metabolite of flavonoids, on which has been reported valuable biological properties and health benefits that, according to the major outcomes described in the present work, would be enclosed to the dietary intake of maqui-citrus beverages [25]. Indeed, DHPAA has been associated with the prevention or delay of glucotoxicity, a key event in the pathogenesis of type II diabetes mellitus, as well as with the protection against pancreatic β-cells dysfunction developed in the frame of hypercholesterolemia [25,26]. This metabolite was found in non-esterified and conjugated forms, being the diglucuronide derivative the only compound exhibiting a significant augment of the urine concentration as a result of the beverages’ intake. The highest urine concentration of non-esterified DHPAA (0.38 µg/mg of creatinine) and the highest augment (85%) corresponded to the ingestion of stevia-sweetened drinks. On the other hand, regarding DHPAA diglucuronide, the highest urine concentration matched with the consumption of beverages prepared using sucrose (0.02 µg/mg of creatinine), as well as the highest percentage augment relative to the basal levels (72%). When analyzing the statistical significance of the augments observed, these corresponded to the consumption of stevia- and sucralose-based beverages (*p* < 0.001) and sucrose-based drinks (*p* < 0.05) for non-esterified DHPAA, and to the daily ingestion of sucralose and sucrose sweetened drinks (*p* < 0.05 and *p* < 0.01, respectively) for DHPAA di-glucuronide.

#### 3.3.3. Eriodictyol, Homoeriodictyol, and Naringenin Derivatives

Eriodictyol (E) is a flavanone present in citrus fruits in a glycosylated form (bound to a rutinoside (Table 1)) and characteristic of lemon. This flavanone was presented in urine non-esterified, as well as bound to sulfate as a result of phase II reactions (E sulfate). Regarding both of them, the highest urine concentration was achieved because of the consumption of sucrose-sweetened beverages (0.01 and 0.09 µg/mg of creatinine, for non-esterified E and E sulfate, respectively). Moreover, non-esterified E experienced a significant augment after the consumption of the maqui-citrus beverages developed using sucrose and sucralose as sweeteners (*p* < 0.001 and *p* < 0.01). However, the intake of stevia-based beverages showed a significant augment of E sulfate (*p* < 0.001) (Figure 1).

In the case of homoeriodictyol (HE) and naringenin (N) derivatives, HE glucuronide and N glucuronide, respectively, were the only metabolites detected. Both increased the urine concentration significantly after the intake of maqui-citrus drinks, independently of the sweetener applied (*p* < 0.001), although the use of sucralose and stevia gave rise to the highest augment for these metabolites. Besides, the sucralose-based beverage provided the highest values for HE glucuronide (2.08 µg/mg of creatinine) and the sucrose-based drink for N glucuronide (142.69 µg/mg of creatinine) (Figure 1).

As referred to before, the flavanones eriodictyol, homoeriodictyol, and naringenin are the most representative (poly)phenols in citrus fruits. Furthermore, these phenolic compounds have been noticed regarding the healthy properties in humans, namely anticancer, antidiabetic, neuroprotective, anti-obesity, anti-inflammatory, cardioprotective, and hepatoprotective, among others [27,28]. In this regard, Liu et al. [29] reported recently that eriodictyol and naringenin can inhibit the formation of advanced glycation end products, closely related to several pathologies like Alzheimer’s disease, neuropathy, retinopathy, or nephropathy [30]. Again, these biological traits further support the modifications of the chemical and phytochemical characteristics of fruit juices that could affect positively the bioavailability of such molecules, and thus the biological effect that could be foreseen.

#### 3.3.4. Trans-Ferulic Acid Derivatives

Concerning trans-ferulic acid (TFA), a phenolic acid formed enzymatically from caffeic acid (CA) in enterocytes, the sulfate derivative was the only phase II metabolite, whose urine concentration augmented significantly for all sweeteners (*p* < 0.01 for stevia- and sucralose-based beverages, and *p* < 0.05 for sucrose-based beverage) [20]. However, although sucralose was the sweetener providing the highest concentration (10.86 µg/mg of creatinine), the intake of stevia-sweetened drinks yielded the highest bioavailability in terms of percentage. This metabolite would be responsible, to some extent, for the biological benefits expected from the consumption ofmaqui-citrus beverages, especially because of its properties against metabolic syndrome [31,32]. Besides, the combination of this phenolic compound with caffeic acid (CA) has been reported as a promising therapy against multiple aspects of this syndrome, as well as against liver steatosis as recently defined through an overweight mouse model [32].

#### 3.3.5. Vanillic Acid Derivatives

Finally, vanillic acid (VA) also showed a significant increase of the urine concentration according to the results retrieved from the paired sample *t*-test applied (*p* < 0.001 for stevia-based drinks, *p* < 0.01 for sucralose-based beverage, and *p* < 0.05 for sucrose-based beverage). Despite the ingestion of the sucrose-based beverages provided the highest values of this metabolite in urine (24.16 µg/mg of creatinine), the intake of beverages developed using stevia as a sweetener, duplicated the basal values, providing the highest increase. This outcome is of special relevance, because of the association of this compound to anti-cancer, anti-obesity, anti-inflammatory, and cardioprotective features [33,34,35].

Overall results referred to the bioavailability of flavanones and anthocyanins metabolites after the intake of the beverages developed indicated that stevia was the most efficient sweetener, in respect to the capacity of the maqui-citrus beverage to raise the basal urine concentration for most metabolites (caffeic acid, 3,4-di-hydroxyphenylacetic acid, eriodictyol sulfate, naringenin glucuronide, trans-ferulic acid sulfate, and vanillic acid), followed by sucralose (caffeic acid glucuronide, caffeic acid sulfate, and homoeriodictyol glucuronide), and sucrose (caffeic acid, 3,4-di-hydroxyphenylacetic acid di-glucuronide, and eriodictyol). These results are in agreement with previous research of the own group, suggesting stevia, a natural non-caloric sweetener, as a valuable alternative to sucrose to reduce sugar intake [13,14]. Furthermore, results showed an increase along the time for most metabolites produced from the (poly)phenolic load of the beverages under consideration, regardless of the significance of the augment and the sweetener employed. This suggests an accumulative effect as a result of the chronic consumption of newly designed beverages. Besides, the healthy properties of the metabolites detected are of special interest due to the biological properties described, which reinforce the protective attributions of the precursor flavanones and anthocyanins against a range of pathophysiological situations.

Moreover, it is important to notice that no differences were observed between BMI at the baseline and 60 days of ingesting maqui-citrus beverages for any of the experimental groups included in the design of the study (mean 28 for all groups at day 0 and 60). These results suggested that there are no differences between sucrose (natural high caloric), stevia (natural non-caloric), and sucralose (artificial non-caloric) in terms of directly modifying the basic nutritional or metabolic status of volunteers. Furthermore, it has to be mentioned that the data from blood samples and from physic parameters (i.e., BMI) would be published somewhere else.

## 4. Conclusions

The results described in the present work evidence a great diversity of metabolites, including phase II derivatives, synthesized due to the metabolism of flavanones and anthocyanins present in maqui-citrus beverages, elaborated from maqui berry and second quality citrus fruits. However, neither parental anthocyanin nor hesperidin aglycones were found. Thus, the major outcomes retrieved suggest stevia as the sweetener that allows obtaining a higher bioavailability for most metabolites of the (poly)phenols present in the newly developed beverages as the basal urine concentrations of these metabolites raised significantly after the ingestion of stevia-sweetened beverages. These metabolites represented all the spectrum of phenolic compounds identified (caffeic acid, 3,4-di-hydroxyphenylacetic acid, eriodictyol, naringenin, trans-ferulic acid, and vanillic acid, except homoeriodictyol) after the beverage intake, regardless of the form (unesterified or phase II derivatives) in which they are detected. Moreover, the beverage sweetened with stevia will group all the healthy properties of the compounds commented above against diseases related to sugar consumption. Concerning sucralose and sucrose, both sweeteners caused the highest bioavailability of coincident metabolites, and thus the efficiency of these sweeteners can be considered as equal. However, the urine concentration values augmented for most derivatives after two months of dietary intervention, independently of the sweetener used, suggesting these metabolites as persistent over time in the volunteers.

As referred above and according to the results retrieved, the use of second quality lemon or other citrus fruits contributes to preventing the incidence and severity of metabolic diseases. Therefore, their inclusion as an ingredient, source of bioactive (poly)phenols, and metabolic derivatives in the newly designed beverages would provide an alternative use for second quality fruits, allowing industries to minimize production losses, along with taking advantage of the benefits for health associated to their (poly)phenolic profile.

Summarizing, when considering the differences in the bioactive compounds bioavailability dependent on the sweetener used, this study suggests stevia and, to a lesser extent, sucralose are valuable alternatives to sucrose, which is directly related to the incidence, prevalence, and severity of type II diabetes mellitus, obesity, and cardiovascular diseases, among other deleterious pathological conditions.

## Figures and Tables

**Table 1:** Flavanone composition (mg/100mL) of the maqui-citrus beverages.

*Flavanone*	Stevia	Sucralose	Sucrose	*p*-Value
O-triglycosyl-N ^Z^	0.18 ± 0.01	0.20 ± 0.01	0.18 ± 0.01	>0.05 ^N.s. Y^
E 7-O-rutinoside	1.95 ± 0.01	1.85 ± 0.01	1.92 ± 0.01	>0.05 ^N.s. Y^
N 7-O-rutinoside	1.73 ± 0.01	1.74 ± 0.01	1.71 ± 0.01	>0.05 ^N.s. Y^
H 7-O-rutinoside	9.09 ± 0.01	9.18 ± 0.01	9.06 ± 0.01	>0.05 ^N.s. Y^
TOTAL	12.95 ± 0.10	12.97 ± 0.13	12.87 ± 0.13	>0.05 ^N.s. Y^

^Z^ N, naringenin; E, eriodictyol; H, hesperetin. ^Y^ N.s., not significant.

**Table 2:** Anthocyanin composition (mg/100mL) of the maqui-citrus beverages.

*Anthocyanin*	Stevia	Sucralose	Sucrose	*p*-Value
Dp 3-O-sam-5-O-glc ^Z^	4.48 ± 0.01	4.48 ± 0.01	4.49 ± 0.01	>0.05 ^N.s. Y^
Dp 3,5-O-diglc	5.13 ± 0.01	5.07 ± 0.01	5.09 ± 0.01	>0.05 ^N.s. Y^
Cy 3-O-sam-5-O-glc + Cy 3,5-O-di-glc	2.07 ± 0.01	2.11 ± 0.01	2.09 ± 0.01	>0.05 ^N.s. Y^
Dp 3-O-sam	1.53 ± 0.01	1.51 ± 0.01	1.49 ± 0.01	>0.05 ^N.s. Y^
Dp 3-O-glc	4.46 ± 0.01	4.48 ± 0.01	4.47 ± 0.01	>0.05 ^N.s. Y^
Cy 3-O-sam	0.53 ± 0.01	0.51 ± 0.01	0.50 ± 0.01	>0.05 ^N.s. Y^
Cy 3-O-glc	0.81 ± 0.01	0.81 ± 0.01	0.80 ± 0.01	>0.05 ^N.s. Y^
TOTAL	19.01 ± 0.22	18.97 ± 0.20	18.93 ± 0.23	>0.05 ^N.s. Y^

^Z^ Cy, cyanidin; Dp, delphinidin; Glc, glucoside; Sam, sambubioside. ^Y^ N.s., not significant.

## Supplementary Materials

The following are available online at https://www.mdpi.com/2072-6643/13/2/312/s1, Table S1: Supplementary Table S1. Proximate composition of the beverages developed using different sweeteners (sucrose, sucralose, and stevia). Table S2: Qualitative analysis of anthocyanin and flavanone metabolites in plasma after the ingestion of maqui-citrus beverages determined by UHLC-ESI-QqQ-MS/MS operated in multiple reaction monitoring, in negative and positive ionization modes, respectively.
