# Supplementary material for: Beverages Based on Second Quality Citrus Fruits and Maqui Berry, a Source of Bioactive (Poly)phenols: Sorting Out Urine Metabolites upon a Longitudinal Study
Source: Nutrients. 2021 Jan 22;13(2):312. doi: 10.3390/nu13020312 (PMC7910882; doi:10.3390/nu13020312)
Supplement: Supplementary file 1 [file nutrients-13-00312-s001.pdf]

Table S1. Proximate composition of the beverages developed using different sweeteners (sucrose, sucralose, and stevia).

Parameter	Sweetener		
	Sucrose	Sucralose	Stevia
Energy (kj/100 mL)	314	123	134
Energy (kcal/100mL)	74	29	32
Total fat (g/100 mL)	0.14	0.22	0.30
Monounsaturated fats (g/100 mL)	0.06	0.10	0.10
Polyunsaturated fats (g/100 mL)	0.06	0.04	0.17
Saturated fats (g/100 mL)	0.02	0.07	0.03
Carbohydrates (g/100 mL)	17.40	6.00	6.10
Total sugar (g/100 mL)	16.90	5.70	6.10
Fructose (g/100 mL)	2.90	1.90	2.10
Glucose (g/100 mL)	2.90	1.80	2.00
Lactose (g/100 mL)	<0.40	<0.40	<0.40
Maltose (g/100 mL)	<0.40	<0.40	<0.40
Sucralose (g/100 mL)	11.10	2.00	2.00
Dietary fiber (g/100 mL)	0.36	0.60	1.00
Ashes (g/100 mL)	0.23	0.23	0.26
Moisture (g/100 mL)	81.30	92.50	91.7
Proteins (g/100 mL)	0.57	0.50	0.66
Salts (g/100 mL)	<0.01	<0.01	<0.01
Sodium (mg/100 mL)	3.10	2.80	2.50

Table S2. Qualitative analysis of anthocyanin and flavanone metabolites in plasma after the ingestion of maqui-citrus juices determined by UHLC-ESI-QqQ-MS/MS operated in multiple reaction monitoring, in negative and positive ionization modes, respectively.

Compound	RT (min)	Precursor ion [M-H]$^-$ *m/z*	Product ion MS2 [M-H]$^-$ *m/z*	Fragmentation (V)	CE (eV)	Polarity
Cyanidin metabolites						
Cyanidin (Cy)	8.81 (N.f.)	287.0	137.0	100	20	Positive
Cy 3-*O*-glucoside	N.f.	449.0	287.0	100	20	Positive
Cy 3,5-*O*-di-glucoside	N.f.	743.0	287.0	100	20	Positive
Cy 3-*O*-sambubioside	N.f.	581.0	287.0	100	20	Positive
Cy 3-*O*-sambubioside-5-*O*-glucoside	N.f.	611.0	287.0	100	20	Positive
Delphinidin metabolites						
Delphinidin (Dp)	5.18 (N.f.)	303.0	229.0/257.0	100	20	Positive
Dp 3-*O*-glucoside	N.f.	465.0	303.0	100	20	Positive
Dp 3,5-*O*-di-glucoside	N.f.	627.0	303.0	100	20	Positive
Dp 3-*O*-sambubioside	N.f.	597.0	303.0	100	20	Positive
Dp 3-*O*-sambubioside-5-*O*-glucoside	N.f.	759.0	303.0	100	20	Positive
Eriodictyol metabolites						
Eriodictyol (E)	6.49	287.0	151.0	70	10	Negative
Eriocitrin	N.f.	449.0	287.0	70	10	Negative
E glucuronide	4.87	463.0	287.0	70	10	Negative
E di-glucuronide	N.f.	639.0	287.0	70	10	Negative
E sulfate	5.53	367.0	287.0	70	10	Negative
E di-sulfate	N.f.	447.0	287.0	70	10	Negative
E glucuronide-sulfate	N.f.	543.0	287.0	70	10	Negative
Hesperetin metabolites						
Hesperetin (H)	7.30 (N.f.)	302.0	151.0	70	20	Negative
Hesperidin	N.f.	609.0	302.0	70	20	Negative
H glucuronide	N.f.	478.0	302.0	70	20	Negative
H di-glucuronide	N.f.	664.0	302.0	70	20	Negative
H sulfate	N.f.	382.0	302.0	70	20	Negative
H di-sulfate	N.f.	462.0	302.0	70	20	Negative
H glucuronide-sulfate	N.f.	558.0	302.0	70	20	Negative
Homoeriodictyol metabolites						
Homoeriodictyol (HE)	7.30 (N.f.)	301.0	151.0	110	15	Negative
HE glucuronide	5.50	477.0	301.0	110	15	Negative
HE di-glucuronide	N.f.	653.0	301.0	110	15	Negative
HE sulfate	N.f.	381.0	301.0	110	15	Negative
HE di-sulfate	N.f.	461.0	301.0	110	15	Negative
HE glucuronide-sulfate	4.67	557.0	301.0	110	15	Negative
Naringenin metabolites						
Naringenin (N)	7.26	271.0	119.0	130	20	Negative
O-triglycosyl-N	4.63	433.0	271.0	130	20	Negative
Narirutin	N.f.	579.0	271.0	130	20	Negative
N glucuronide	5.07	433.0	271.0	130	20	Negative
N di-glucuronide	N.f.	623.0	271.0	130	20	Negative
N sulfate	N.f.	351.0	271.0	130	20	Negative
N di-sulfate	N.f.	431.0	271.0	130	20	Negative
N glucuronide-sulfate	N.f.	527.0	271.0	130	20	Negative
Caffeic acid metabolites						
Caffeic acid (CA)	3.25 (N.f.)	179.1	135.0	70	15	Negative
CA glucuronide	2.40	355.1	179.1	70	15	Negative
CA di-glucuronide	1.67	531.1	179.1	70	15	Negative
CA sulfate	2.99	259.1	179.1	70	15	Negative
CA glucuronide-sulfate	1.95	435.1	179.1	70	15	Negative
CA di-Sulfate	N.f.	339.1	179.1	70	15	Negative

Catechol metabolites						
Catechol (CAT)	5.04 (N.f.)	109.0	67.0	80	6	Negative
CAT glucuronide	N.f.	286.0	109.0	80	6	Negative
CAT di glucuronide	2.83 (N.f.)	461.0	109.0	80	6	Negative
CAT sulfate	1.59 (N.f.)	189.0	109.0	80	6	Negative
CAT glucuronide-sulfate	1.38 (N.f.)	365.0	109.0	80	6	Negative
CAT di-sulfate	N.f.	269.0	109.0	80	6	Negative
3,4-Dihydroxyphenylacetic acid metabolites						
3,4-Dihydroxyphenylacetic acid (DHPAA)	1.80	166.8	123.2	70	5	Negative
DHPAA glucuronide	1.58	342.8	166.8	70	5	Negative
DHPAA di-glucuronide	1.04	518.8	166.8	70	5	Negative
DHPAA sulfate	1.14	246.8	166.8	70	5	Negative
DHPAA glucuronide-sulfate	0.74	422.8	166.8	70	5	Negative
DHPAA di-sulfate	1.07	326.8	166.8	70	5	Negative
Hippuric acid metabolites						
Hippuric acid (HA)	2.55	178.0	134.4	80	5	Negative
HA glucuronide	1.70 (N.f.)	354.0	178.0	80	5	Negative
HA di-glucuronide	0.59 (N.f.)	530.0	178.0	80	5	Negative
HA sulfate	1.78	258.0	178.0	80	5	Negative
HA glucuronide-sulfate	1.50	434.0	178.0	80	5	Negative
HA di-sulfate	N.f.	338.0	178.0	80	5	Negative
Gallic acid metabolites						
Gallic acid (GA)	0.71 (N.f.)	169.0	125.0	70	10	Negative
GA glucuronide	N.f.	345.0	169.0	70	10	Negative
GA di-glucuronide	N.f.	521.0	169.0	70	10	Negative
GA sulfate	N.f.	249.0	169.0	70	10	Negative
GA glucuronide-sulfate	N.f.	425.0	169.0	70	10	Negative
GA di-sulfate	N.f.	329.0	169.0	70	10	Negative
Trans-*ferulic acid metabolites*						
Trans-ferulic acid (TFA)	4.46	192.8	133.8	20	5	Negative
TFA glucuronide	4.25	368.8	192.8	20	5	Negative
TFA di-glucuronide	1.74 (N.f.)	544.8	192.8	20	5	Negative
TFA sulfate	3.56 (N.f.)	272.8	192.8	20	5	Negative
TFA glucuronide-sulfate	N.f.	448.8	192.8	20	5	Negative
TFA di-sulfate	1.32	352.8	192.8	20	5	Negative
2,4,6-Trihydrobenzaldehid metabolites						
2,4,6-Trihydrobenzaldehid (THBA)	5.10	153.1	106.8	90	18	Negative
THBA glucuronide	5.08 (N.f.)	329.1	153.1	90	18	Negative
THBA di-glucuronide	N.f.	505.1	153.1	90	18	Negative
THBA sulfate	1.46	233.1	153.1	90	18	Negative
THBA glucuronide-sulfate	N.f.	409.1	153.1	90	18	Negative
THBA di-sulfate	N.f.	313.1	153.1	90	18	Negative
Trans-isoferulic acid metabolites						
Trans-isoferulic acid (TIFA)	1.46	193.7	134.7	70	5	Negative
TIFA glucuronide	N.f.	366.7	193.7	70	5	Negative
TIFA di-glucuronide	N.f.	545.7	193.7	70	5	Negative
TIFA sulfate	1.45 (N.f.)	273.7	193.7	70	5	Negative
TIFA glucuronide-sulfate	N.f.	449.7	193.7	70	5	Negative
TIFA di-sulfate	N.f.	353.7	193.7	70	5	Negative
Vanillic acid metabolites						
Vanillic acid (VA)	3.18	167.0	151.8	100	15	Negative
VA glucuronide	1.57 (N.f.)	343.0	167.0	100	15	Negative
VA di-glucuronide	1.01	519.0	167.0	100	15	Negative
VA sulfate	1.14	247.0	167.0	100	15	Negative
VA glucuronide-sulfate	0.93	423.0	167.0	100	15	Negative
VA di-sulfate	1.13 (N.f.)	327.0	167.0	100	15	Negative

N.f., not found
